# A nationwide survey of hydroxychloroquine retinopathy presenting to the hospital eye service in the United Kingdom

**DOI:** 10.1038/s41433-022-02291-0

**Published:** 2022-11-15

**Authors:** Imran H. Yusuf, Ruofan C. Han, Susan M. Downes, Srilakshmi M. Sharma

**Affiliations:** 1 Nuffield Laboratory of Ophthalmology, Nuffield Department of Clinical Neurosciences, University of Oxford, West Wing, John Radcliffe Hospital, Headley Way, Oxford, OX3 9DU UK; 2 Oxford Eye Hospital, John Radcliffe Hospital, Oxford University Hospitals NHS Foundation Trust, Headley Way, Oxford, OX3 9DU UK

**Keywords:** Retinal diseases, Eye manifestations

## Abstract

**Background:**

The risk of developing hydroxychloroquine retinopathy is considered sufficient to justify national monitoring programmes. There are an estimated 71,144–77,170 long-term hydroxychloroquine users in the UK. However, the number of patients diagnosed with retinopathy is unknown. This study aimed to identify the number of cases and clinical characteristics of hydroxychloroquine retinopathy diagnosed annually in hospital eye services across the UK.

**Methods:**

A nationwide, prospective case ascertainment study was undertaken using the British Ophthalmological Surveillance Unit, which sends approximately 1420 reporting cards to UK Ophthalmologists monthly. The case definition was two abnormal tests suggestive of hydroxychloroquine retinopathy. Demographic and clinical data relating to hydroxychloroquine use and retinopathy were collected from identified cases using a standardised questionnaire over a 1-year period (2018–2019).

**Results:**

Sixty-six cases of hydroxychloroquine retinopathy were reported, and 46 questionnaires were received (73% response rate). Twenty-four incident cases of hydroxychloroquine retinopathy were identified (24-43 cases following adjustment). The median duration of drug therapy was 19 years (range: 4–50 years, IQR: 14.5–23 years). Fourteen patients were asymptomatic, and 9 symptomatic at diagnosis. A trend towards a lower mean deviation on visual field testing was observed in the symptomatic group (−11.55 dB versus −6.9 dB; *P* = 0.15).

**Conclusion:**

Between 1 in 1655 and 3215 (0.03–0.06%) long-term hydroxychloroquine users were diagnosed with retinopathy over the study period. We estimate that monitoring was available for 1.9–3.8% of long-term users, accounting for a lower than expected incidence. The high proportion of symptomatic retinopathy at diagnosis underlines the importance of monitoring to detect pre-symptomatic disease.

## Introduction

Hydroxychloroquine retinopathy is a toxic retinopathy that can result in severe and progressive visual loss following exposure to hydroxychloroquine [1]. Prescriptions of hydroxychloroquine have more than doubled in the past 10 years in the United Kingdom [2], which coupled with low drug discontinuation rates [3, 4], has led to significant numbers of patients on long-term hydroxychloroquine who are at risk of retinopathy.

The advent of modern retinal imaging techniques—in particular, optical coherence tomography and fundus autofluorescence imaging—has enabled the detection of pre-symptomatic retinopathy [1, 5]. Using these techniques, Melles and Marmor (2014) identified retinopathy in 7.5% of patients with more than 5 years of exposure to hydroxychloroquine, increasing to 20–50% after 20 years based on a single abnormal test result consistent with hydroxychloroquine retinopathy [6]. Jaumouille et al identified a prevalence of 6.5% in long-term users using the same definition of toxicity [7]. A large, single-centre prospective audit of 869 long-term hydroxychloroquine users who underwent monitoring in the UK identified a prevalence of 6.3% if one abnormal test result was used to define toxicity, and 1.6% if two abnormal tests were used to define toxicity [8]. The Royal College of Ophthalmologists (RCOphth) recommendations specify that two abnormal tests suggestive of toxicity are required to diagnose definite retinopathy (2018 and 2020) [9, 10].

Monitoring for hydroxychloroquine retinopathy has been recommended by the American Academy of Ophthalmology (AAO) since 2002 [11] and the RCOphth since 2018 [10]. Despite these recommendations, which have been adopted in many countries, it is unclear how many individuals are diagnosed with hydroxychloroquine retinopathy each year. This is the first national survey of hydroxychloroquine retinopathy cases seen by the UK hospital eye service. Data presented in this paper include patients reported for the first year (July 2018–2019) of a 2-year reporting period. The primary aim of the study was to determine the number of cases of hydroxychloroquine retinopathy presenting to the hospital eye service across the UK, enabling an estimate of the frequency of detected retinopathy in long-term users and an overview of hydroxychloroquine monitoring coverage in the UK. The secondary aims were to identify the demographics of confirmed cases, clinical features, retinal imaging characteristics, dosing characteristics, risk factors for retinopathy and the management of hydroxychloroquine retinopathy.

## Methods

### Study design

The study was undertaken from July 2018 to July 2019 as a British Ophthalmological Surveillance Unit (BOSU) of the Royal College of Ophthalmologists (RCOphth) study. Screening - or monitoring, as is the preferred term in the UK since the disorder is a known adverse effect of a prescribed drug - for hydroxychloroquine retinopathy was recommended by the RCOphth 5 months prior to the start of this study [10]. The overall population coverage of monitoring at the time was considered to be low as services were being organised. Hence, the study was implemented at a time when both symptomatic and asymptomatic patients with hydroxychloroquine retinopathy would be identified, allowing a comparison between the groups. The study was approved by a national research ethics committee in the United Kingdom (17/SC/0574) and adhered to the tenets of the Declaration of Helsinki. The study questionnaire was further scrutinised by the BOSU research committee.

All permanent consultant and associate specialist ophthalmologists (approximately 1420 ophthalmologists) in the United Kingdom were notified via the British Ophthalmological Surveillance Unit (BOSU) of the diseases which should be reported on a monthly basis. On notification of a case, the study investigators requested that the reporting ophthalmologist completed a questionnaire for each case (Supplementary Fig. 1). This was then returned directly to the study investigators for analysis. The study was additionally advertised through the Medical Retina Group (MRG), a UK-based group of Consultant Ophthalmologists with expertise in medical retina. Reminders were sent to reporters who did not return the study questionnaire in order to increase the response rate.

**Fig. 1:**
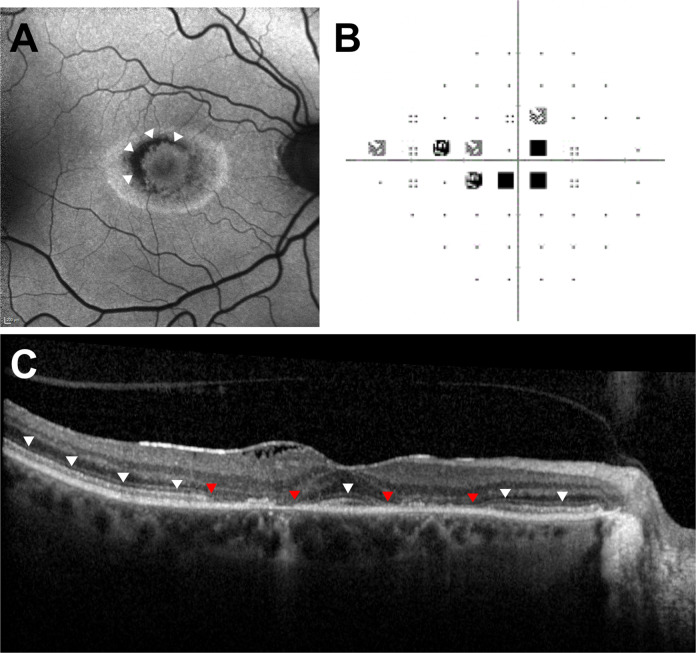
Retinal imaging studies of a patient with symptomatic hydroxychloroquine retinopathy. **A** Short-wavelength fundus autofluorescence imaging demonstrates a classical parafoveal distribution of hydroxychloroquine retinopathy with sparing of the fovea. The focal decrease in signal in a concentric ring around the fovea (arrowheads) indicates loss of the retinal pigment epithelium; **B** Automated visual field testing (24-2) plot demonstrates a central scotoma congruous to the area of RPE loss. **C** Optical coherence tomography imaging demonstrates thinning of the outer nuclear layer with disruption and loss of the external limiting membrane (ELM) (red arrowheads indicate the limits of ELM loss) in the parafovea, and preservation of the ELM at the fovea and more peripherally (white arrowheads). There is an epiretinal membrane.

The case definition was described as follows: “macular dysfunction due to hydroxychloroquine toxicity confirmed on at least one investigation of the following: automated visual field, OCT, autofluorescence or electrodiagnostic testing”. The study questionnaire collected case details relating to the demographics of each reported case, date of diagnosis, ocular co-morbidities, primary treatment indication, the presence of symptoms at diagnosis, visual acuity, automated Humphrey visual field test results, tests used to support a diagnosis of hydroxychloroquine retinopathy, including external limiting membrane (ELM) status on spectral-domain optical coherence tomography at diagnosis (see Fig. 1), disease distribution on retinal imaging, as well as drug dosing characteristics (daily dose and duration of therapy), risk factors (renal impairment, tamoxifen use), and initial management of hydroxychloroquine retinopathy.

### Reference denominator value

The reference denominator value of UK hydroxychloroquine users is 153,659 to 166,673 individuals, with the number of long-term (>5 years) users estimated at 71,144–77,170.

These values are derived from two different methodologies:

#### Clinical practice research datalink (CPRD)

The CPRD estimated that 166,673 individuals take hydroxychloroquine in the UK, with 46.3% of hydroxychloroquine users remaining on the drug after 5 years (predicting 77,170 long-term hydroxychloroquine users) [12].

#### NHS digital

Data derived from the NHS Digital Prescription Cost Analysis database suggests 153,659 hydroxychloroquine users in the UK. This value is derived from an annual 1,213,295 pack sales of 60 × 200 mg tablets, followed by adjustments for national average daily dose (313 mg) and UK population. These figures are in line with published estimates using a similar methodology [2]. Estimates of drug continuation rates predict approximately 71,144 long-term hydroxychloroquine users.

### Data analysis

Statistical analysis was performed using GraphPad Prism version 8.3.1 (GraphPad Software, California, USA). Normality of all datasets were assessed using Shapiro-Wilk. Non-parametric data were compared using the Mann-Whitney test for unpaired and Wilcoxon matched-pairs signed rank test for paired comparisons, as appropriate. Descriptive statistics of non-parametric data are expressed as median and interquartile range. Parametric data were compared using *t*-test with Welch’s correction, which accounts for variation in the standard deviation between datasets. Descriptive statistics of parametric data are presented as means with standard deviations. Contingency analysis was undertaken using the Fischer’s exact test. The threshold for statistical significance was *P* < 0.05.

The specific statistical tests applied to the data were as follows. The frequency of hydroxychloroquine retinopathy presenting to the Hospital Eye Service was expressed as a percentage of long-term hydroxychloroquine users in the UK as approximated to the two reference denominator values of 71,144–77,170 users, presented above. The raw value of reported cases within the 1-year study interval was adjusted to account for both under-ascertainment and under-reporting, presented as a range against the two reference denominator values.

Demographic data, clinical characteristics and dosing characteristics of the cohort of incident hydroxychloroquine retinopathy cases were represented as counts, medians, and interquartile ranges. Visual acuity (logMAR best corrected visual acuity) and visual fields (mean deviation in decibels) were non-parametric variables and represented with counts, medians and interquartile ranges. Patient age was considered as a continuous parametric variable, and was represented with mean, range, and standard deviation. Symptom and ELM status at diagnosis were considered dichotomous variables, and were also represented as counts and percentages.

A comparison of measures of visual function (e.g. visual acuity and visual field parameters) were first compared between right and left eyes using Wilcoxon matched-pairs signed rank test. For all subsequent analysis, the eye with the poorest visual function in each patient was used for comparison between individuals. Subgroup analysis was performed examining visual acuity, mean visual field deviation, duration of HCQ treatment, HCQ dosage (non-parametric data, Mann–Whitney test) and patient age (parametric data, Welch’s unpaired *t*-test) between symptomatic and asymptomatic patients, and those with and without ELM disruption on OCT imaging at diagnosis.

## Results

### Frequency of hydroxychloroquine retinopathy presenting to the hospital eye service in long-term users

**Fig. 2:**
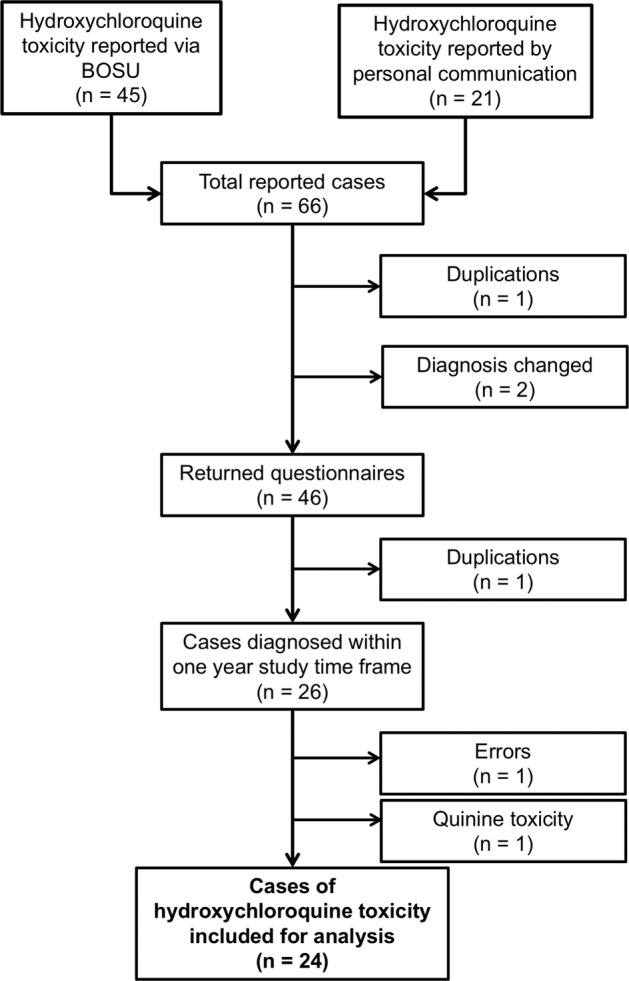
Flow of case recruitment and exclusion. BOSU British Ophthalmological Surveillance Unit.

Over a 1-year study period, 66 cases of hydroxychloroquine toxicity were reported through BOSU or by personal communication to the investigators (Fig. 2). One case was excluded due to duplication of reporting. Personal communication was received regarding 2 unreturned questionnaires where the diagnosis had changed. Sixty-three study questionnaires were sent to reporting consultants, of which 46 were returned (73% response rate). After exclusion of a further case of duplication, a case of quinine toxicity, a case of erroneous reporting and cases which received their diagnosis outside the study period of 1 July 2018 to 1 July 2019 (*n* = 20), 24 incident cases of hydroxychloroquine retinopathy were identified during the study period (Fig. 2). Adjusting for the under-ascertainment (76% BOSU report card return rate) and under-reporting (73% response rate), the potential number of cases of hydroxychloroquine retinopathy presenting to the hospital eye service during the study year was between 24 and 43 cases.

Twenty-three of the 24 reported patients (96%) had taken hydroxychloroquine for more than 5 years. Using a denominator value of 71,144–77,170 long-term hydroxychloroquine users in the UK, these data suggest that approximately 1 in 1655–3215 (0.03-0.06%) long-term hydroxychloroquine users were diagnosed with retinopathy by the hospital eye service in the UK in the year of study.

### Demographic characteristics of reported cases

Complete datasets were obtained for 19 patients and partial datasets for 5 patients. Demographic information, clinical parameters and dosing characteristics of reported patients are summarised in Table 1. In the cohort, there were 23 females and 2 males. The most common indication for hydroxychloroquine use was SLE (*n* = 12), followed by RA (*n* = 6), Sjögren’s syndrome (*n* = 5), mixed connective tissue disease (*n* = 1) and malaria (*n* = 1). 75% of individuals were White British (*n* = 17), with Black Caribbean (*n* = 3), Other White (*n* = 2), Other Black (*n* = 1), and Irish (*n* = 1).

**Table 1:** Summary of patient characteristics.

**Patient characteristics**				
Median visual acuity at diagnosis, logMAR (range, IQR)	**OD**		**OS**	
	0.0 (−0.1 to 0.5, 0–0.2)		0.02 (−0.1 to 0.32, −0.02 to 0.2)	
Median 10-2 Humphrey visual field deficit at diagnosis, dB (range, IQR)	−3.73 (−29.96 to −1.81, −8.52 to −3.21)		−2.77 (−24.48 to −0.30, −7.55 to −2.05)	
Disease distribution	**Parafoveal**	**Pericentral**		**Mixed**
	18	2		3
Median daily dose, mg (range, IQR)	200 (57–400, 200–400)			
Median duration of use, years (range, IQR)	19 (4–50, 14.5–23)			
ELM disruption on OCT imaging at diagnosis	15 (63%)			
**Comparison of symptomatic and asymptomatic patients**				
	**Symptomatic**		**Asymptomatic**	
Number	9 (39%)		14 (61%)	
Mean age, years (range, SD)	55.9 (30–82, 15.8)		57.8 (28-86, 17.4)	
Median duration of use, years (range, IQR)	15 (4–25, 13-21)		20 (7-50, 15.25–24.25)	
Median daily dose, mg (range, IQR)	238.4 (57.1–400, 200–400		277.6 (200–400, 200–400)	
Median visual acuity of worst seeing eye, logMAR (range, IQR)	0.02 (-0.1 to 0.5, 0 –0.2)		0 (−0.1 to 0.3, 0–0.19)	
Median field deficit of worst seeing eye at diagnosis, dB (range, IQR)	−7.26 (-29.96 to −3.15, −15.85 to −4.03)		-3.51 (-24.48 to −1.81, −6.09 to −2.81)	
**Disease distribution**				
Parafoveal	5		8	
Pericentral	1		2	
Mixed	2		0	
ELM disruption on OCT imaging at diagnosis	7 (78%)		7 (50%)	
**Comparison of patients based on ELM status on OCT at diagnosis**				
	**ELM disruption at diagnosis**		**ELM preserved at diagnosis**	
Number	15		9	
Mean age in years (range, SD)	55 (28–86, 46.5–72)		55 (30–71, 36–62)	
Median visual acuity of worst seeing eye, logMAR (range, IQR)	0.14 (0.5 to −0.1, 0.02–0.3))		0.0 (0.2 to −0.1, −0.1 to 0)	
Median field deficit of worst seeing eye at diagnosis, dB (range, IQR)	-13.55 (−29.96 to −1.81, −22.90 to −4.88)		−3.51(−12.82 to −2.22, −4.28 to −3.11)	
Median duration of use, years (range, IQR)	15.5 (10–5, 15–20.1)		20(4–40, 8–25)	
Median daily dose, mg (range, IQR)	200 (57.1–400, 200–400)		200 (200–400, 200–400)	

*IQR* interquartile range, *dB* decibels, *SD* standard deviation.

**Fig. 3:**
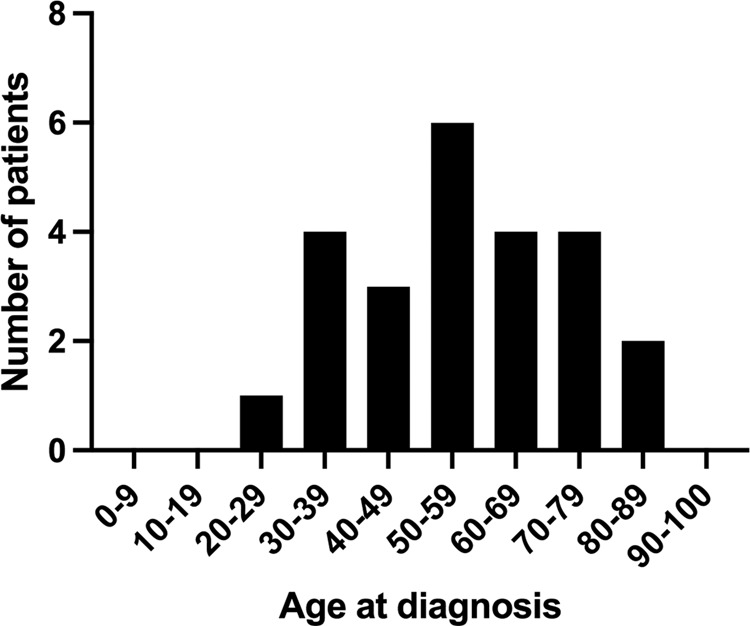
Age distribution of patients with hydroxychloroquine retinopathy at diagnosis. The mean age at diagnosis was 56.3 years (standard deviation of 17.5 years).

The age distribution at diagnosis of patients reported in this study are presented in Fig. 3.

### Hydroxychloroquine dosing characteristics

Data relating to daily dose and duration of hydroxychloroquine use were not normally distributed within the cohort. The median daily hydroxychloroquine dose was 262 mg (IQR: 200–400 mg). Although most patients were taking 200 mg or 400 mg per day, some patients were on variable dosing regimens. The median duration of hydroxychloroquine therapy was 19 years (IQR: 14.5–23 years).

### Clinical characteristics

Data relating to logMAR visual acuity and visual field parameters were not normally distributed within the cohort. Median visual acuity at diagnosis was logMAR 0.0 for the right and 0.02 for the left eyes (IQR right 0–0.2, left -0.02-0.2). Median 10-2 visual field sensitivity at diagnosis was −3.73 dB in right eyes and -2.77 dB in left eyes (IQR right: −8.52 dB to −3.21 dB, left: −7.55 dB to −2.05 dB). There was no significant difference in visual acuities (*p* = *0.91*) or visual field sensitivities (*p* = *0.1*; Wilcoxon matched-pairs signed rank test for both tests), between the right and left eyes. For subsequent analyses we used visual acuity and visual field data from the worse-seeing eye for each patient. 14 patients underwent automated 10–2 visual field testing, and 3 patients, automated 24-2 visual field testing. Due to the small number of patients with 24–2 field data, these were excluded from statistical analysis of visual field data.

96% of patients (23/24) had at least 2 abnormal tests of the following testing types: fundoscopy, Humphrey visual field, fundus autofluorescence and optical coherence tomography: 76% had at least 3 abnormal tests (16 out of 21). 6 patients went on to have an ERG of which 4 were abnormal; 5 had a multifocal ERG, all of which were reported as abnormal. 57% of patients had disease identifiable on fundoscopy (13/23), and 100% of patients had abnormal visual fields. 92% had abnormal SD-OCT imaging (22/24) and 77% had an abnormal fundus autofluorescence imaging test result (17/22). 63% of patients had ELM disruption at diagnosis (*n* = 15). The ratio of paracentral: pericentral disease: mixed disease was 18:2:3.

### Symptomatic versus asymptomatic patients

**Fig. 4:**
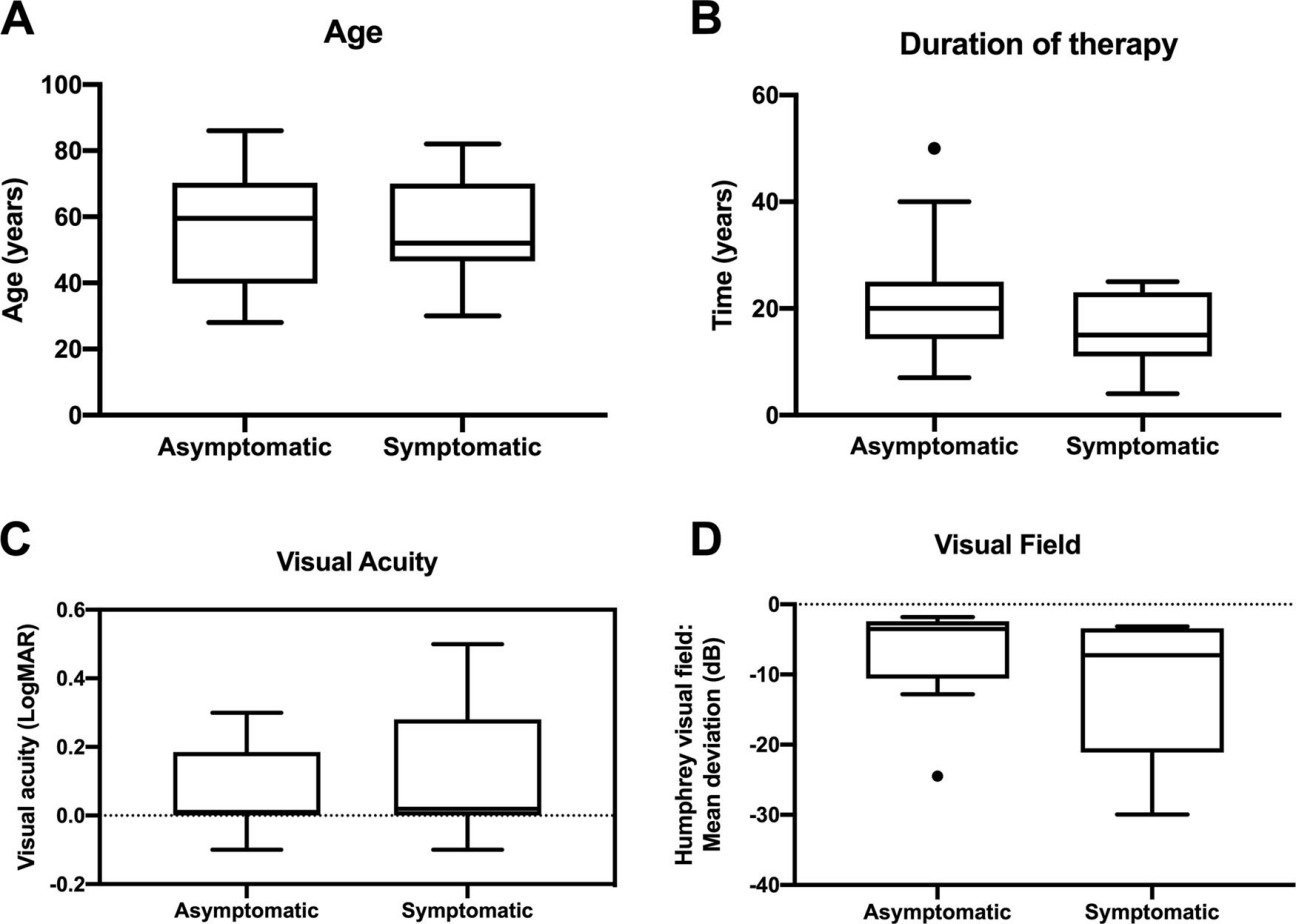
Comparison of asymptomatic and symptomatic hydroxychloroquine retinopathy patients. **A** Age (median of 59.5 years versus 52 years; *p* = 0.79, Welch’s *t*-test). **B** Duration of therapy (median of 20 years versus 15 years; *p* = 0.29, Mann–Whitney test). **C** Visual acuity (median logMAR 0.00 versus 0.06; *p* = 0.51; Welch’s *t*-test). **D** Visual field (median mean deviation −3.5 dB versus −7.26 dB; *p* = 0.23, Mann–Whitney test). All box plots present median values, interquartile ranges and minimum and maximum values for each chart. The Tukey method was used to exclude outliers which are indicated as points on the relevant box plots.

The ratio of asymptomatic to symptomatic patients at diagnosis was 14:9 (data missing for one male patient). Of the symptomatic patients, 56% complained of worse visual acuity (one patient had both scotoma and reduced visual acuity and has been counted in both groups, and one further patient had both reduced visual acuity and micropsia and has been counted in both groups) (*n* = 5), 22% of scotoma (*n* = 2), 22% of a deficit in colour vision (*n* = *2*), and 33% had ‘other’ symptoms including glare and photopsia (*n* = 3). We found no statistically significant differences in patient age (*p* = 0.79; Welch’s unpaired *t*-test, parametric, Fig. 4A), duration of hydroxychloroquine usage (*p* = 0.29; Mann–Whitney unpaired *t*-test, non-parametric, Fig. 4B) visual acuity (*p* = 0.51; Welch’s unpaired t-test, parametric, Fig. 4C), visual field sensitivity (*p* = 0.23; Mann–Whitney unpaired *t*-test, non-parametric, Fig. 4D), hydroxychloroquine dose (*p* = 0.52; Mann–Whitney unpaired *t*-test, non-parametric), or the number of patients with ELM disruption (*p* = 0.13; Fischer’s exact contingency test for dichotomous variables of symptom and ELM disruption status) between the symptomatic and asymptomatic patient groups.

### External limiting membrane (ELM) status on optical coherence tomography imaging

We performed a subgroup analysis of patients with ELM disruption on OCT imaging compared to those with a preserved ELM at diagnosis (Supplementary Fig. 2). There was no statistically significant difference in age (*p* = 0.30; Welch’s unpaired *t*-test, parametric), duration of hydroxychloroquine usage (*p* = 0.82, Mann–Whitney unpaired t-test, non-parametric) hydroxychloroquine dose (*p* = 0.94, Mann–Whitney unpaired *t*-test, non-parametric), or presence or absence of symptoms *(p* = 0.23, Mann–Whitney unpaired *t*-test, non-parametric) between patients based on ELM status. However, patients with ELM preservation at diagnosis had significantly better visual acuity (median logMAR of 0.0; IQR 0.1; non-parametric) than patients with ELM disruption (median logMAR of 0.14; IQR 0.28; non-parametric) (*p* = 0.002; Mann–Whitney unpaired *t*-test, non-parametric). Although there was no statistically significant difference in visual field sensitivity between patients with ELM disruption or ELM preservation at diagnosis (*p* = 0.18; Mann–Whitney unpaired *t*-test, non-parametric), there appeared to be a trend towards better visual field status in patients with a preserved ELM (median MD value: −3.51 dB; IQR −2.1 dB) (median MD value: −13.55 dB; IQR −22.75 dB) (Supplementary Fig. 2).

### Management

Eighty-three percent (*n* = 20) of patients were managed with cessation of hydroxychloroquine at diagnosis. 13% (*n* = 3) were continued at the same dose pending rheumatological review (data unavailable for one case).

## Discussion

These data suggest that approximately 1 in 1655 to 1 in 3215 (0.03–0.06%) of all long-term hydroxychloroquine users were diagnosed with retinopathy in the hospital eye service annually in the UK, during the study interval. This range represents the limits of error following adjustment for case under-ascertainment and under-reporting. On a population level, the incidence of hydroxychloroquine retinopathy in the UK is approximately 0.36–0.65 cases per million per year (Supplementary Table 1).

Marshall et al. identified a frequency of definite retinopathy of 1.6% in a UK cohort of 869 long-term hydroxychloroquine retinopathy users who underwent monitoring procedures using the RCOphth criteria for definite toxicity of two abnormal tests consistent with retinopathy [8]. Ophthalmologists reporting cases in this study used the same definition of hydroxychloroquine retinopathy. The frequency of retinopathy between 0.03–0.06% of long-term users is far lower than those presented in recent cohort studies [6, 7]. There are a number of reasons for this. Firstly, previous studies defined the prevalence of retinopathy in a cohort long-term users in which all individuals received retinal imaging and visual field testing, rather than an estimated 1.9–3.8% in this study. Secondly, there is an approximate four-fold reduction in the diagnostic yield in the same cohort when a definition of toxicity of two abnormal tests is used, rather than one abnormal test [8]. Thirdly, this study sought only incident cases where a new diagnosis was made strictly within the one-year study period, not prevalent cases, as reported in most other studies.

Although the hydroxychloroquine retinopathy risk characteristics of the estimated 71,144 to 77,170 long-term hydroxychloroquine users in the UK are unknown, a frequency of retinopathy of 1.6% suggests that, with universal coverage of retinal monitoring, approximately 1146–1243 individuals in the population may satisfy the criteria for “definite retinopathy”. The identification of 24-43 cases of hydroxychloroquine retinopathy in the UK hospital eye service suggests that the overall monitoring coverage in the UK was between 1.9% and 3.8% during the study interval. This low estimate is consistent with the finding in this survey that ~40% of individuals diagnosed with hydroxychloroquine retinopathy in the hospital eye service were symptomatic at diagnosis, and were therefore unlikely to be detected as a result of monitoring procedures, either before or after the RCOphth recommendations. A repeat study using identical methodology would enable the coverage of monitoring to be reassessed at a future date.

Monitoring for hydroxychloroquine retinopathy has two objectives: (1) to detect retinopathy at an early stage prior to symptomatic presentation, (at which point visual function is likely to be better preserved); (2) to detect retinopathy at a stage in which disease will not progress. There are currently no studies that directly evaluate whether patients detected through monitoring have better visual function than those presenting with symptoms. Since hydroxychloroquine retinopathy spares the fovea until at an advanced stage, visual acuity was not significantly different between the symptomatic and asymptomatic groups. However, the average automated visual field mean deviation was worse in the group presenting with visual symptoms (average mean deviation: −11.55 dB) when compared to the asymptomatic group (average mean deviation: −6.9 dB), although this was not statistically significant (*p* = 0.15). Based on the measured effect size of visual field mean deviation between the groups (0.53), a total sample size of 124 would be required in order to have a 90% power to detect a difference between these groups. This study was not designed to assess the efficacy of monitoring: further studies are required to prove definitively that patients without symptoms have better visual function and potentially a better prognosis than symptomatic patients at diagnosis.

Preservation of the external limiting membrane (ELM) on OCT imaging has been identified as a positive prognostic indicator in hydroxychloroquine retinopathy that predicts a lower likelihood of disease progression [13, 14]. ELM integrity has also been considered an important predictive sign of visual acuity for other macular disorders [15–17]. In this study, we identified a significantly worse visual acuity in patients with OCT evidence of ELM disruption at the time of diagnosis of hydroxychloroquine retinopathy. Further studies are required to investigate the role of the ELM in hydroxychloroquine retinopathy.

The British Ophthalmological Surveillance Unit uniquely permits the capture of rare diseases by UK Consultant Ophthalmologists (approximately 1420 individuals). This study design may not be applicable to other settings, for example where allied healthcare professionals may diagnose retinopathy. However, despite this, it is expected that all new cases of hydroxychloroquine retinopathy, even if diagnosed in monitoring programmes outside the hospital service, will be referred for an Ophthalmology appointment enabling capture of these cases.

This study has several limitations. Firstly, the diagnosis of hydroxychloroquine retinopathy was made by reporting clinicians without a mechanism of verification by the study investigators. However, the clinicians were experienced (i.e. Consultant Ophthalmologists) who reported “definite” retinopathy as defined by the RCOphth such that “possible” or very mild cases were not included. Secondly. statistical adjustments for under-ascertainment and response rate were necessary, presenting a margin of error to the disease estimates—these are methodological limitations largely inherent to all BOSU studies. Thirdly, the first year of data capture was underpowered to detect a difference between symptomatic and asymptomatic groups which may be achieved across the 2-year study. Fourthly, the denominator reference value of hydroxychloroquine users in the UK is based on dispensed tablets (NHS Digital), rather than consumed tablets. However, estimates using the CPRD which aggregates data from primary care converge on similar values. The range between these estimates are represented by limits of error as stated in the subsequent calculations.

The increasing use of hydroxychloroquine in developed countries in the past two decades [2] and low drug discontinuation rates [3, 4] is likely to increase the incidence of hydroxychloroquine retinopathy in the future given the latent period between treatment initiation and the development of retinopathy. Increasing access to retinal monitoring using modern retinal imaging techniques will facilitate the detection of early manifestations of hydroxychloroquine retinopathy.

In summary, this national survey suggests that approximately 1 in 1655 to 1 in 3215 (0.03–0.06%) of all long-term hydroxychloroquine users were diagnosed with retinopathy by hospital eye services annually in the UK during the study interval. These data suggest that retinal monitoring for long-term hydroxychloroquine users had a coverage of between 1.9% and 3.8% during the study interval, thus highlighting the need to widen access to retinal screening and monitoring in this patient group.

## Summary

### What was known before

- Hydroxychloroquine retinopathy is more common than previously thought, affecting approximately 7.5% of those taking the drug for more than 5 years.
- The incidence of disease has not been identified in the UK.
- The population coverage of retinal screening is unknown in the UK.

### What this study adds

- We identified 24 individuals diagnosed with hydroxychloroquine retinopathy in the UK. over a 1-year period
- These data suggest that approximately 0.03–0.06% of the ~70,000 long-term hydroxychloroquine users in the UK were diagnosed with retinopathy over the study interval.
- These data suggest that the approximate coverage of retinal screening at the time of the study was less than 4% in long-term hydroxychloroquine users. As a result, approximately 40% of those diagnosed were symptomatic at diagnosis (i.e. had advanced disease).

## Supplementary information

Supplementary Table 1

Supplementary Figure 1

Supplementary Figure 2

Description of Additional Supplementary Files

## Data Availability

The datasets generated during and/or analysed during the current study are available from the corresponding author on reasonable request.
