# Supplementary material for: A nationwide survey of hydroxychloroquine retinopathy presenting to the hospital eye service in the United Kingdom
Source: Eye (Lond). 2022 Nov 15;37(10):2082–8. doi: 10.1038/s41433-022-02291-0 (PMC10333228; doi:10.1038/s41433-022-02291-0)
Supplement: Supplementary file 1 — Supplementary Table 1 [file 41433_2022_2291_MOESM1_ESM.docx]

| **Long-term (>5 years) hydroxychloroquine users in the UK** | **Unadjusted proportion of reported cases**  **(24 cases)** | **Frequency** |  | **Adjusted proportion of reported cases**  **(24-43 cases)** | | **Frequency** | |
| --- | --- | --- | --- | --- | --- | --- | --- |
|  |  |  |  | *Min* | *Max* | *Min* | *Max* |
| 71,144 | 0.034% | 1 in 2964 |  | 0.034% | 0.060% | 1 in 2964 | 1 in 1655 |
| 77,170 | 0.031% | 1 in 3215 |  | 0.031% | 0.056% | 1 in 3215 | 1 in 1795 |

| **Frequency of retinopathy in UK reference cohort**  **(Marshall *et al*, 2021)** | **Expected number of individuals with retinopathy in the UK based on two estimates** | | **Coverage of monitoring services in the UK, based on adjusted number of reported cases** | |
| --- | --- | --- | --- | --- |
|  |  |  | *Min* | *Max* |
| 1.6% | Estimate 1: 71,144 | 1146 | 2.1% | 3.8% |
|  | Estimate 2: 77,170 | 1243 | 1.9% | 3.5% |

| **Raw population-based incidence of reported cases, adjusted** | **UK population, 2018** | **Overall population frequency** | **Cases per million per year** |
| --- | --- | --- | --- |
| 24 *(lower limit)* | 66,460,000 | 1 in 2,769,167 | 0.36 |
| 43 *(upper limit)* |  | 1 in 1,545,581 | 0.65 |
