# Supplementary material for: A nationwide survey of hydroxychloroquine retinopathy presenting to the hospital eye service in the United Kingdom
Source: Eye (Lond). 2022 Nov 15;37(10):2082–8. doi: 10.1038/s41433-022-02291-0 (PMC10333228; doi:10.1038/s41433-022-02291-0)
Supplement: Supplementary file 2 — Supplementary Figure 1 [file 41433_2022_2291_MOESM2_ESM.docx]

1. **Case details**

Patient local hospital number: ________________

Month and year of birth: ____________________

Patient Sex: 🞏 Male 🞏 Female

| **White** | **Asian or Asian British** | **Black or Black British** | **Chinese** | **Mixed Race** | **Other ethnic group** |
| --- | --- | --- | --- | --- | --- |
| ❑ British  ❑ Irish  ❑ Other (please specify) | ❑ Indian  ❑ Pakistani  ❑ Bangladeshi  ❑ Other (please specify) | ❑ Caribbean  ❑ African  ❑ Other (please specify) | ❑ Chinese  ❑ Other (please specify) | ❑ (please specify)  ____________ | ❑ Other (please specify) |

***Here are some questions about the diagnosis of hydroxychloroquine toxicity in this patient***

1. **What was the date of confirmation of the diagnosis of hydroxychloroquine retinopathy?**

(Short case definition: macular dysfunction due to hydroxychloroquine toxicity confirmed on one investigation out of: automated visual field, OCT, autofluorescence or electrodiagnostics). For full case definition see Page 3 of this document.

___________/__________/__________ *[DD/MM/YYYY]*

**3. Does the patient have any relevant ocular co-morbidities?**

☐ Cataract

☐ Diabetic maculopathy

☐ Glaucoma

☐ Age related macular degeneration

☐ Choroidal neovascularisation

☐ Macular oedema associated with vein occlusion

☐ Other, please specify:

1.______________________________ 2.______________________________ 3._____________________________

☐ Don’t know

***Next, we have some questions about indications and symptoms:***

**4. What is the patient’s primary treatment indication for hydroxychloroquine?**

☐ Systemic Lupus Erythematosus

☐ Rheumatoid Arthritis

☐ Other disorder (including dermatological). Please specify: ______________________________

☐ Don’t know

**5. At diagnosis, what symptoms did the patient have?**

☐ Scotoma

☐ Reduced visual acuity

☐ Deficit in colour vision

☐ Other: please specify __________________________________

☐ Don’t know

☐ Patient was asymptomatic

***Next are some questions about the abnormalities on investigation of the patient***

**6. Vision at diagnosis**

|  | RIGHT EYE | LEFT EYE |
| --- | --- | --- |
| **Best corrected visual acuity**  (Snellen or LogMAR) |  |  |
| **Visual field Mean Deviation**  (in decibels (dB))  Please circle visual field protocol:  10-2 / 24-2 / 30-2 / not done |  |  |

**7. What investigations were used to diagnose hydroxychloroquine retinopathy in this patient?** Please tick a column for each investigation result in this case.

| **Investigation** | **Normal** | **Abnormal** | **Not needed** | **Not available** |
| --- | --- | --- | --- | --- |
| Fundoscopy |  |  |  |  |
| Humphrey visual field |  |  |  |  |
| Fundus autofluorescence |  |  |  |  |
| Spectral domain OCT |  |  |  |  |
| Electrodiagnostic testing: ERG |  |  |  |  |
| Electrodiagnostic testing:  Multifocal ERG |  |  |  |  |

**8. Was the external limiting membrane disrupted on SD-OCT imaging at diagnosis?**

☐ Yes

☐ No

**9. What distribution of hydroxychloroquine retinopathy existed in this patient?**

**Please select A, B or C**

| **A** | **B** | **C** |
| --- | --- | --- |
| **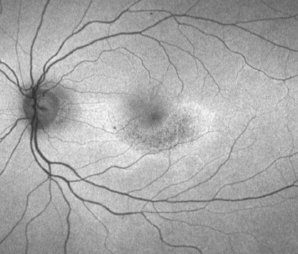** | **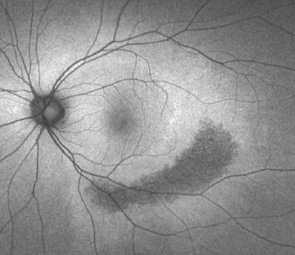** | **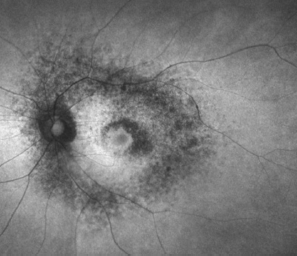** |
| **Paracentral disease** | **Pericentral disease** | **Mixed: paracentral and pericentral disease** |
| retinal changes within a disc  diameter of the foveal centre | retinal changes > 1 disc diameter  away from the foveal centre | Paracentral AND pericentral  distribution of disease |

**10. How long had this patient been taking hydroxychloroquine for?**

__________ years _________months

☐ Don’t know

**11. What was the daily dose of hydroxychloroquine when the diagnosis was made?**

__________ milligrams per day

☐ Don’t know

**12. Did this patient have known renal impairment?**

☐ Yes

☐ No

☐ Don’t know

**13. Did this patient take tamoxifen in the past or at the time of diagnosis?**

☐ Tamoxifen taken previously

☐ Tamoxifen taken at the time of diagnosis of hydroxychloroquine retinopathy

If known, what was the duration of tamoxifen use: __________years

☐ No history of tamoxifen use

☐ Don’t know

**14. How was this patient with hydroxychloroquine retinopathy managed at diagnosis?**

☐ Hydroxychloroquine treatment was stopped

☐ Hydroxychloroquine dose was reduced: the new dose was ________mg per day

☐ Hydroxychloroquine was continued at the same dose

☐ Don’t know

**15. If hydroxychloroquine was continued, please specify the reason for this:**

☐ Don’t know

**16. Was this patient referred to your ophthalmology department from another ophthalmology department?**

☐ Yes. Please specify hospital referred from:________________________________

☐ No

☐ Don’t know

**Thank you for taking the time to complete this questionnaire.**

Please return questionnaire to: Mr Imran Yusuf, Specialist Registrar in Ophthalmology, Oxford Eye Hospital, West Wing, John Radcliffe Hospital, Oxford, OX3 9DU.

Incomplete questionnaires should also be returned if outstanding clinical details cannot be identified.
