# Supplementary figures and images for: A nationwide survey of hydroxychloroquine retinopathy presenting to the hospital eye service in the United Kingdom
Source: Eye (Lond). 2022 Nov 15;37(10):2082–8. doi: 10.1038/s41433-022-02291-0 (PMC10333228; doi:10.1038/s41433-022-02291-0)

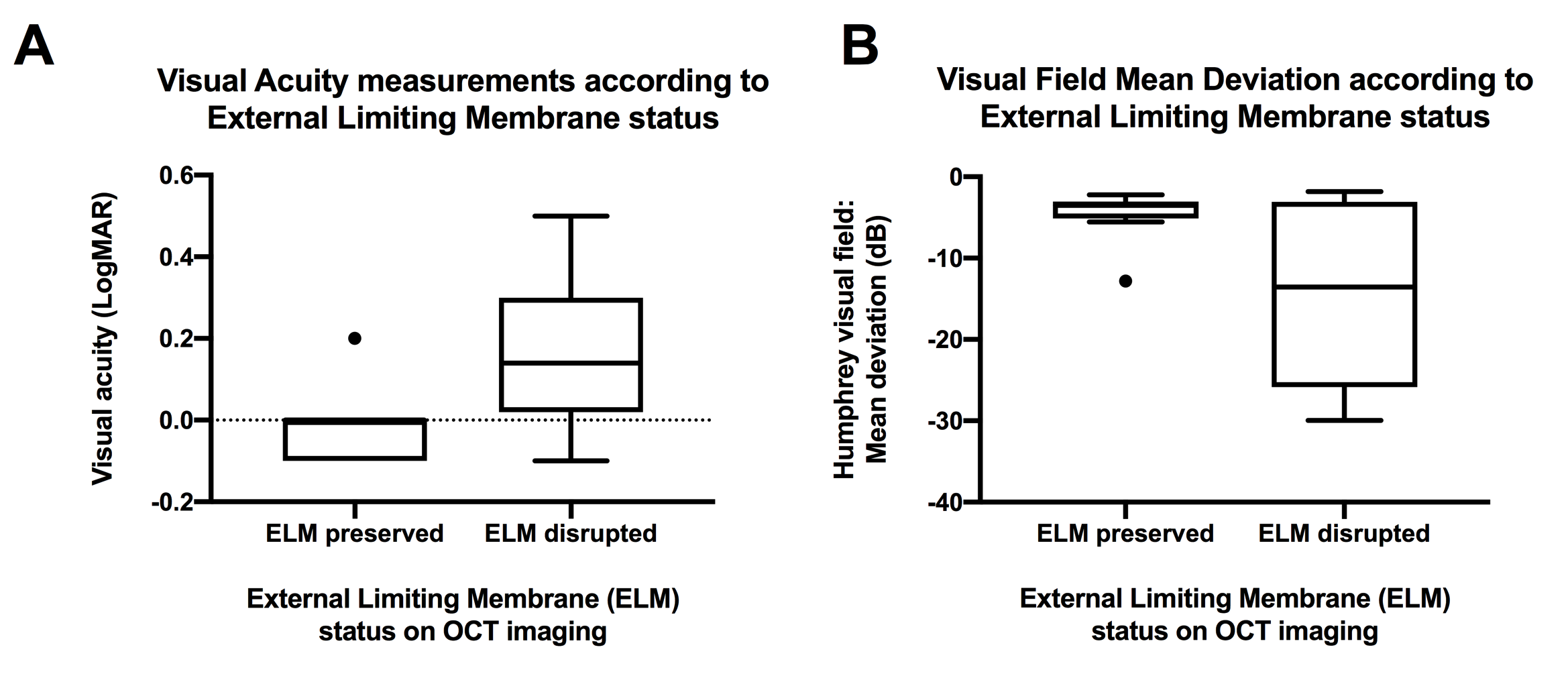

Supplement: Supplementary file 3 — Supplementary Figure 2 [file 41433_2022_2291_MOESM3_ESM.tif]
