# Supplementary material for: A nationwide survey of hydroxychloroquine retinopathy presenting to the hospital eye service in the United Kingdom
Source: Eye (Lond). 2022 Nov 15;37(10):2082–8. doi: 10.1038/s41433-022-02291-0 (PMC10333228; doi:10.1038/s41433-022-02291-0)
Supplement: Supplementary file 4 — Description of Additional Supplementary Files [file 41433_2022_2291_MOESM4_ESM.docx]

**Supplementary Figure 1.** The study questionnaire used to collect data presented in this study.

**Supplementary Figure 2.** Analysis of hydroxychloroquine retinopathy patients according to external limiting membrane (ELM) status on optical coherence tomography imaging at diagnosis. **(a)** Visual acuity (median LogMAR acuity of 0.00 in ELM preserved and 0.14 in ELM disrupted group; *p=0.002,* Mann-Whitney test) **(b)** Visual field (median mean deviation of -3.4dB in ELM preserved group versus -13.55dB in ELM disrupted group; *p=0.18*, Mann-Whitney test). All box plots present median values, interquartile ranges and minimum and maximum values for each chart. The Tukey method was used to exclude outliers, which are indicated as points on the relevant box plots.

**Supplementary Table 1.** A summary of derivations of disease estimates.
